# High Throughput Screening Identifies Novel Lead Compounds with Activity against Larval, Juvenile and Adult *Schistosoma mansoni*

**DOI:** 10.1371/journal.pntd.0004659

**Published:** 2016-04-29

**Authors:** Nuha R. Mansour, Ross Paveley, J. Mark F. Gardner, Andrew S. Bell, Tanya Parkinson, Quentin Bickle

**Affiliations:** 1 Department of Immunology and Infection, London School of Hygiene and Tropical Medicine, London, United Kingdom; 2 Salvensis, Discovery Park House, Discovery Park, Sandwich, Kent, United Kingdom; McGill University, CANADA

## Abstract

An estimated 600 million people are affected by the helminth disease schistosomiasis caused by parasites of the genus *Schistosoma*. There is currently only one drug recommended for treating schistosomiasis, praziquantel (PZQ), which is effective against adult worms but not against the juvenile stage. In an attempt to identify improved drugs for treating the disease, we have carried out high throughput screening of a number of small molecule libraries with the aim of identifying lead compounds with balanced activity against all life stages of *Schistosoma*. A total of almost 300,000 compounds were screened using a high throughput assay based on motility of worm larvae and image analysis of assay plates. Hits were screened against juvenile and adult worms to identify broadly active compounds and against a mammalian cell line to assess cytotoxicity. A number of compounds were identified as promising leads for further chemical optimization.

## Author Summary

Schistosomiasis is a parasitic infection that affects an estimated 600 million people in developing countries. Treatment is currently dependent on a single drug which is only active against the adult form of the disease. Treatment of infected patients eliminates adult worms but leaves any juveniles to survive and develop, thus continuing the infection. A drug acting equally against adult and juvenile worms is likely to be more effective at eliminating the disease. We have identified quality chemical leads with balanced activity against all life stages of the parasite and with a good window over cytotoxicity, which could form the basis of an exciting new drug discovery project.

## Introduction

Schistosomiasis is a potentially severe helminthic disease causing widespread morbidity and affecting an estimated 600 million people [1]. Humans are infected percutaneously by the cercarial larval stage shed from aquatic snails and thereafter the worms remain in the human blood stream migrating from the skin, through the lungs and maturing in the liver. The adult worms then migrate to the mesenteric veins or the vesical plexus of the bladder, depending on species, where they lay eggs, which cause inflammation and fibrosis in various organs.

Praziquantel (PZQ) is the only drug recommended for treatment of schistosomiasis and its increasingly widespread use in mass chemotherapy campaigns means it is the mainstay of control of this infection [2]. PZQ has proved to be generally safe and effective using a single oral dose [3, 4], however reliance on a single drug has led to concerns over potential development of drug resistance. Although PZQ is active against adult worms of all the medically important *Schistosoma* species [5], it is relatively ineffective against the juvenile stages both *in vivo* and *in vitro* [6, 7] meaning that treatment does not eliminate all the worms in an infected patient and repeat treatment is required. Consequently there is a need for new schistosomicides with activity against all life stages and this has led to renewed interest in research into drug discovery using both phenotypic and target-based approaches. These include development and application of whole organism screens for compound testing [8], target-based drug discovery [9] and identification of putative molecular targets by analysis of the annotated schistosome sequences [10].

We have developed a high-throughput screen (HTS) based on use of the larval, schistosomula, stage for primary screening of large compound libraries [11] and here we describe its use as part of an *in vitro* screening program prior to application of medicinal chemistry optimization. Our ultimate aim is to identify compounds with better activity against the juvenile worms than PZQ but with at least comparable activity against the adult worms. Whilst it was unlikely that high-throughput screening hits would be as efficacious as an established drug candidate, selection of hits with multi-stage efficacy is likely to give the best chance of developing a clinical candidate with the desired profile. Therefore all hits were subsequently screened against juvenile (3 week old) schistosomes and adult worms as well as against mammalian cells to check for cytotoxicity. We here report the screening of almost 300,000 small molecule compounds provided from pharmaceutical companies, charitable organizations and commercial sources and describe a number of promising new leads for further chemical optimization.

## Methods

### Ethics Statement and Animals

Experimentation was carried out using the NC3Rs and ARRIVE guidelines under the United Kingdom Animal’s Scientific Procedures Act 1986 (under project licence 60/4456) with approval from the London School of Hygiene and Tropical Medicine Ethics committee. Male CD1 mice (aged 5–6 weeks) were bred on site using SPF conditions with access of food and water *ab libitum*.

### Parasite Maintenance and Preparation

Experiments were performed using the Puerto Rican strain of *S*. *mansoni* maintained in *Biomphalaria glabrata* and CD1 mice. The strain of snail was one bred by Prof Mike Doenhoff (Nottingham University) for high susceptibility to *S*. *mansoni* and ease of breeding in large numbers to high density. Careful attention was paid to controlling infections with rotifers which otherwise would contaminate the HTS schistosomula preparations as well as affecting snail viability. This included recovery of egg masses on short length pieces of polypropylene tubing placed into the breeding tanks and treatment of these with 3 x 10 seconds immersion in 70% ethanol with 3 minutes soaking in clean water in between alcohol treatments. Schistosomula were mechanically prepared as previously described [12] with the modification of using a 45% and 70% Percoll gradient, and the cercarial heads recovered from the 70% layer. All centrifugation steps were done at 350x *g*. The schistosomula generated were then incubated overnight in M169 supplemented with 100U/ml Penicillin, 300μg/ml Streptomycin, 0.25μg/ml Fungizone (Amphotericin B) (Gibco, UK) and 5% Foetal Calf serum.

For production of *S*. *mansoni* juvenile or adult worms, mice were infected subcutaneously under mild isoflurane (Merial Animal Health Ltd (UK)) anaesthesia with, respectively 1400 (for juveniles) or 450 cercariae (for adults). Worms were recovered from infected mice using sterile techniques by portal perfusion 3 weeks (for juveniles) or 6 weeks (for adults) post-infection using warm perfusion medium (Dulbecco’s Modified Eagle’s Medium [DMEM], 2mM L-glutamine, 100 Units/ml penicillin, 100μg/ml streptomycin, 20mM Hepes, 10 Units/ml heparin [Sigma, UK] [8]. Adult worms were washed free of red blood cells using the perfusion medium, and finally placed in culture in complete medium (cDMEM: DMEM, 2mM L-glutamine, 100 Units/ml penicillin, 100μg/ml streptomycin, 10% foetal calf serum (FCS) at 37°C, in an atmosphere of 5% CO_2_. Juvenile worms were sedimented successively following washing with cold perfusion medium and then suspended in cold cDMEM until dispensed to avoid attachment to the plastic tubes.

### *Schistosoma* Screening Assays

The larval HTS was run as described previously [11]. Compound libraries were provided in 384 well plates as liquid either (i) small volumes in polypropylene 384 V-bottom storage plates (Greinerbio-one) that were diluted and 500nL dispensed into black 384 imaging plates (ViewPlate 6007460; PerkinElmer) using the Biomek FxP (Beckman Coulter) or (ii) in an assay-ready format from which a 40nL volume was transferred into wells of imaging plates. All test plates included the same array of control wells: medium alone [4 wells], medium plus DMSO carrier (negative control) [16 wells], 10μM Oltipraz (OLT) positive control [8 wells] or 10μM Praziquantel (PZQ) [4 wells]. Through the interactive reporting system the larval images for all control wells and wells defined as hits by the algorithm were reviewed manually to confirm viability/hit status. Periodically HTS wells suffered from yeast contamination that could not be eliminated by modifications in snail rearing and more extensive washing of the schistosomula preparations. Therefore, to prevent fungal contamination 0.25μg/ml (0.27μM) of amphotericin B (AMP-B) was added to cultures both during the overnight culture prior to plating and during the 3 day assay. This concentration of AMP-B is below the concentrations (1–10μM) reported to have lethal activity against *S*. *mansoni* schistosomula [13, 14]. In our assays the mean ± SD phenotype and motility scores for the DMSO control larvae before and after implementation of use of AMP-B showed minimal differences. The phenotype scores were not significantly different by Student t-test and motility scores showed marginally higher values for AMP-B treated plates (P<0.004). The mean values for the PZQ control wells used in all screening plates were slightly lower in the presence of AMP-B for phenotype but not for motility (phenotype: -AMP-B: -0.21 ± 0.12 and +AMP-B: -0.38 ± 0.08 (P<0.001); motility: -AMP-B: -0.53 ± 0.10 and +AMP-B: -0.57 ± 0.10).

For the juvenile assay the concentration of worms was adjusted to 40/ml cDMEM and, prior to dispensing, the suspension was cooled on ice to prevent adherence of the worms to the plastic bottles. After swirling to resuspend worms,150μL of suspension was added to the wells of 96 well microtitre plates (Nunc, Thermo Fisher Scientific, UK). Wells were quickly checked and those with <5 worms were marked. The remainder were made up to 200μL and the deficient wells topped up with a worm suspension to ensure that all wells had ≥5 worms. The adult worm assay was as described in [8] except that 1ml cultures containing 3 worm pairs were generally used. Both the juvenile and adult worm assays were assessed for activity on days +1 and +5. Drug effects were determined by assessing the viability of individual worms and calculating the mean percentage inhibition, a hit being defined as ≥70% reduction in viability.

### Cytotoxicity Assay

Activity against mammalian cells was determined using MRC-5 cells [15]. Cells were maintained in Minimum Essential Medium supplemented with glutamax (Gibco, UK), 1% non-essential amino acids (Gibco, UK) and 10% Foetal bovine serum (Gibco, UK). They were harvested by treatment with 0.25% Trypsin-EDTA for 3 minutes and diluted to 1x10^5^/ml. 100μl aliquots of the cell suspension were then plated in 96 well plates (Nunc, Thermo Fisher Scientific, UK). The plates were left to incubate for 24 hours at 37°C and 5% CO_2_ in a humidified incubator. Cytotoxicity DR assays were set up 24 hours post incubation at 5% CO_2_ and 37°C, with compounds prepared at 3-fold dilutions starting from 50μM. Dilutions were added to the cells such that the final % DMSO/well was 0.5%. Each drug concentration was tested in duplicate. On each 96 well plate there were negative control wells of media only, MRC-5 cells only and MRC-5 cells with 0.5% DMSO. For positive control wells doxorubicin hydrochloride (Sigma-Aldrich, UK) was added to the cells at 100μM concentration. The cultures were then maintained for a further 72 hours at 37°C and 5% CO_2_. Drug cytotoxicity was determined using the redox indicator Alamar blue (AbD Serotec, UK). 10μl of Alamar Blue was added to each well and the plates incubated for 4 hours at 37°C and 5% CO_2_ atmosphere. The fluorescence intensity was measured using a Spectramax Gemini plate reader (Molecular Devices, UK) using an excitation wavelength of 530 nm and an emission wavelength of 580 nm.

### Screening Sequence

**Fig 1:**
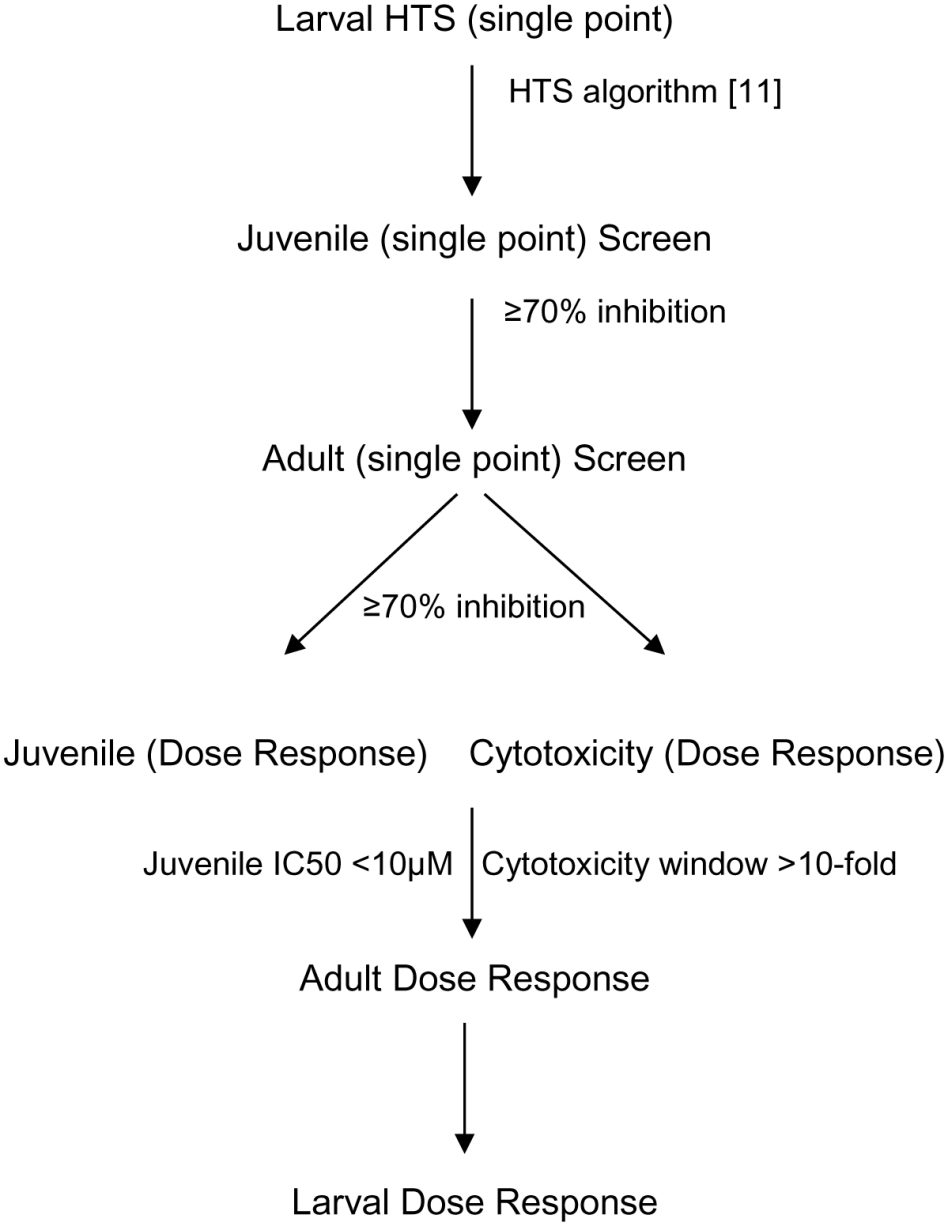
Screening sequence for HTS and follow up.

The screening sequence is shown in Fig 1. The primary HTS and subsequent juvenile and adult screens were run as single point (SP) assays at 10–12.5μM depending on the concentrations in the compound collections. Compounds were tested in duplicate. Where necessary, repeat testing was carried out on re-supplied liquid samples. All compounds of interest were re-tested as solid compounds or by repeat synthesis and activity confirmed.

Compounds with activity against all three parasite stages were tested in parallel dose response (DR) assays against juvenile worms and MRC-5 cells. Promising compounds were then tested in the adult DR. For the parasite DR (larvae, juveniles and adults) the concentrations tested were two fold dilutions starting from 2x the primary screening concentrations (i.e. 20–25μM).

### Data Analysis

IC50 values were calculated from the drug concentration–response curves using Microsoft XLfit version 5.1.0.0 (2006–2008 ID Business Solutions Ltd). Results were expressed as the geometric mean IC50.

### Compound Collections

Compounds were obtained from four sources. The MMV collection was obtained in 96 well plates as 2mM solutions in DMSO and was a diverse chemical library. The Pfizer collection was obtained as 4mM solutions in DMSO and comprised a set of compounds that had previously shown activity against a variety of pathogens. The European Screening Port collection of structurally diverse compounds was obtained in 384 well plates as 2mM solutions in DMSO from European Screening Port GmbH, Schnackenburgallee 114, D-22525 Hamburg (European Screening Port is now Fraunhofer Institute for Molecular Biology and Applied Ecology, http://www.ime.fraunhofer.de/en.html). The published TCAMS collection of compounds with activity against the malaria parasite *Plasmodium falciparum* was provided by GSK (www.ebi.ac.uk/chemblntd, [16]) as 1mM solutions in DMSO. For the larger compound sets from Enamine and MMV, we had the opportunity to select plates of compounds. In doing so we were able to bias the selections we screened towards rule-of-5 like properties [17] and also to select plates containing compounds such that the overall selection was quite diverse. Indeed, the final screened collections contained no duplicate compounds.

**Fig 2:**
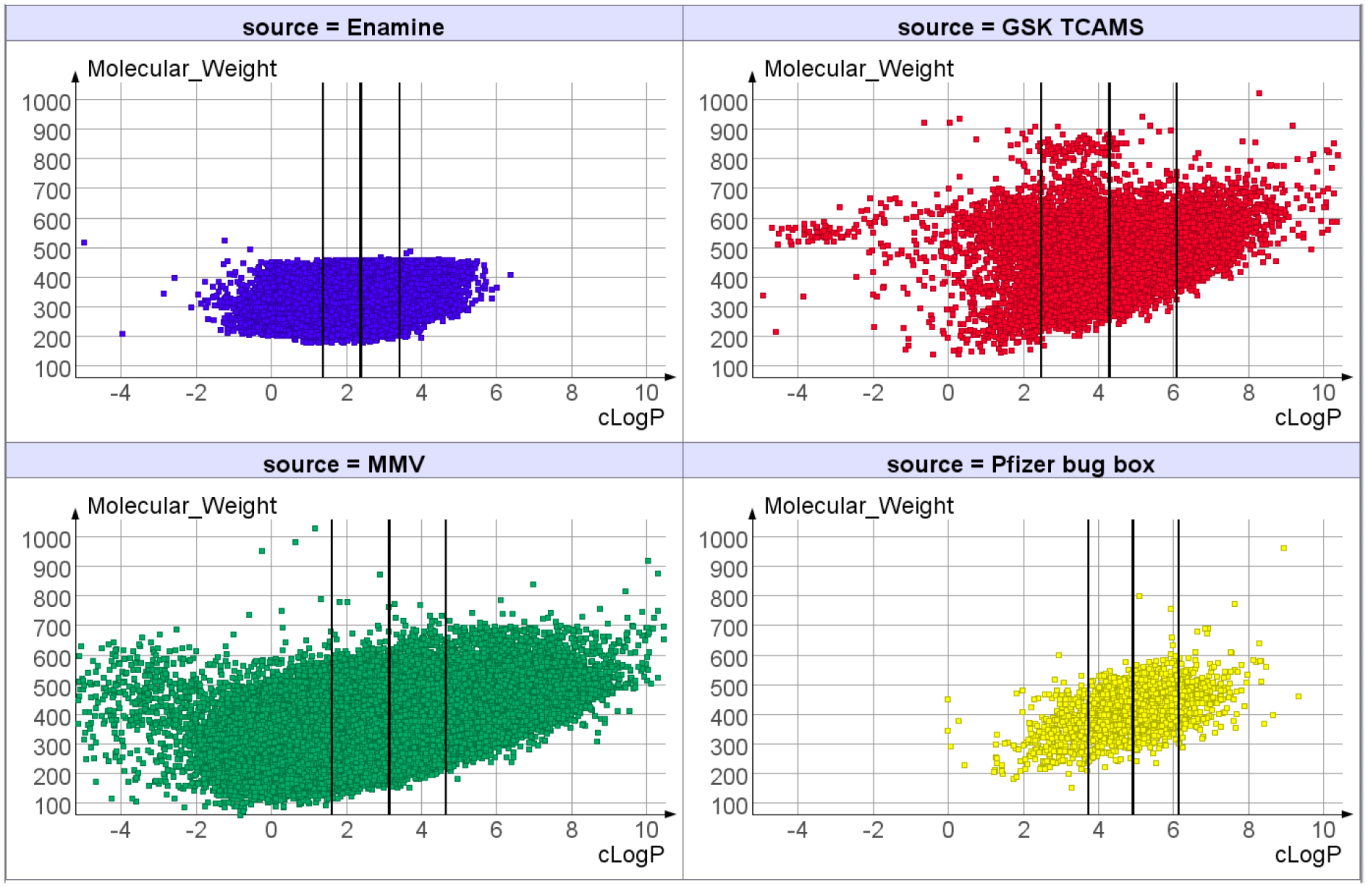
Scatter plots of the screened compound collections with calculated lipophilicity, cLogP using DataWarrior [25] on the X axis vs molecular weight on the Y axis.

The chemical diversity of the compound collections was further investigated by comparing scatter plots (Fig 2). Although there is significant overlap in chemical property space, the scatter plots also demonstrate some differences between them.

## Results

### HTS

Overall 294,253 compounds were tested in the HTS. Table 1 shows the mean Z’ factors for the plates in each of the different compound collections along with the mean phenotype and motility scores for the OLT and the PZQ control wells. The mean Z’ factors for both the phenotype and motility scores were consistently above 0.5 and essentially similar both within and between the different libraries, indicating that this is a robust assay. Similarly the mean phenotype and motility scores for both OLT and PZQ were broadly consistent within and between the different collections and as expected based on previous screening [11]. As was reported previously [11], OLT phenotype and motility scores were consistently higher than those for PZQ indicating a more severe effect of this compound on the worms.

**Table 1:** 

Library	MMV	Pfizer	Enamine	GSK
No. Plates	695	22	151	41
Z’ Phenotype	0.55 ±0.06	0.56 ±0.08	0.53 ±0.07	0.51 ±0.07
Z’ Motility	0.60 ±0.07	0.62 ±0.09	0.59 ±0.07	0.57 ±0.07
PZQ Phenotype score*	-0.37 ±0.09	-0.37 ±0.04	-0.35 ±0.09	-0.50 ±0.10
PZQ Motility score*	-0.57 ±0.10	-0.63 ±0.07	-0.52 ±0.08	-0.67 ±0.12
OLT Phenotype score*	-0.55 ±0.06	-0.56 ±0.08	-0.49 ±0.13	-0.64 ±0.06
OLT Motility score*	-0.95 ±0.02	-0.94 ±0.02	-0.91 ±0.04	-0.93 ±0.03

*NB the mean negative control (Medium plus DMSO carrier) values for the assays are adjusted to zero as described [11]

The hit rates in the primary larval screen varied markedly for the different compound libraries ranging from 0.58% to almost 30% (Table 2). Given the consistent performance of the HTS throughout the screening campaign and the fact that some of the collections were screened in parallel, it is likely that the differences reflect the nature of the compound collections rather than any variation in the performance of the assay.

**Table 2:** Hit rates for compound collections.

Library	No compounds	% (no.) larval hits	% (no.) juvenile hits*	% (no.) adult hits **
MMV	221,948	5.52(12264)	10.17 (1210)	5.12 (62)
Pfizer	7,688	29.89 (2362)	12.52 (126)	19.84 (25)
Enamine	51,392	0.58 (300)	4.33 (13)	38.46 (5)
GSK	13,225	29.09 (3848)	19.33 (58)	15.55 (9)

Larval, juvenile and adult hits were defined as >70% inhibition

* percentage of larval hits tested which were juvenile hits

**percentage of juvenile hits tested which were adult hits

N.B. Due to drug availability issues, pre-existing cytotoxicity data, compound structure or cost, not all hits from any stage were necessarily screened in successive screens.

High numbers of cercariae were used for the infection of mouse donors meaning that the juvenile assay could be run in medium throughput and provided an important triage step in the screening sequence. It was observed that the smaller juvenile worms generally proved to be more susceptible to compound inhibition than the larger worms.

### Active Compounds Selected for Further Follow Up

**Fig 3:**
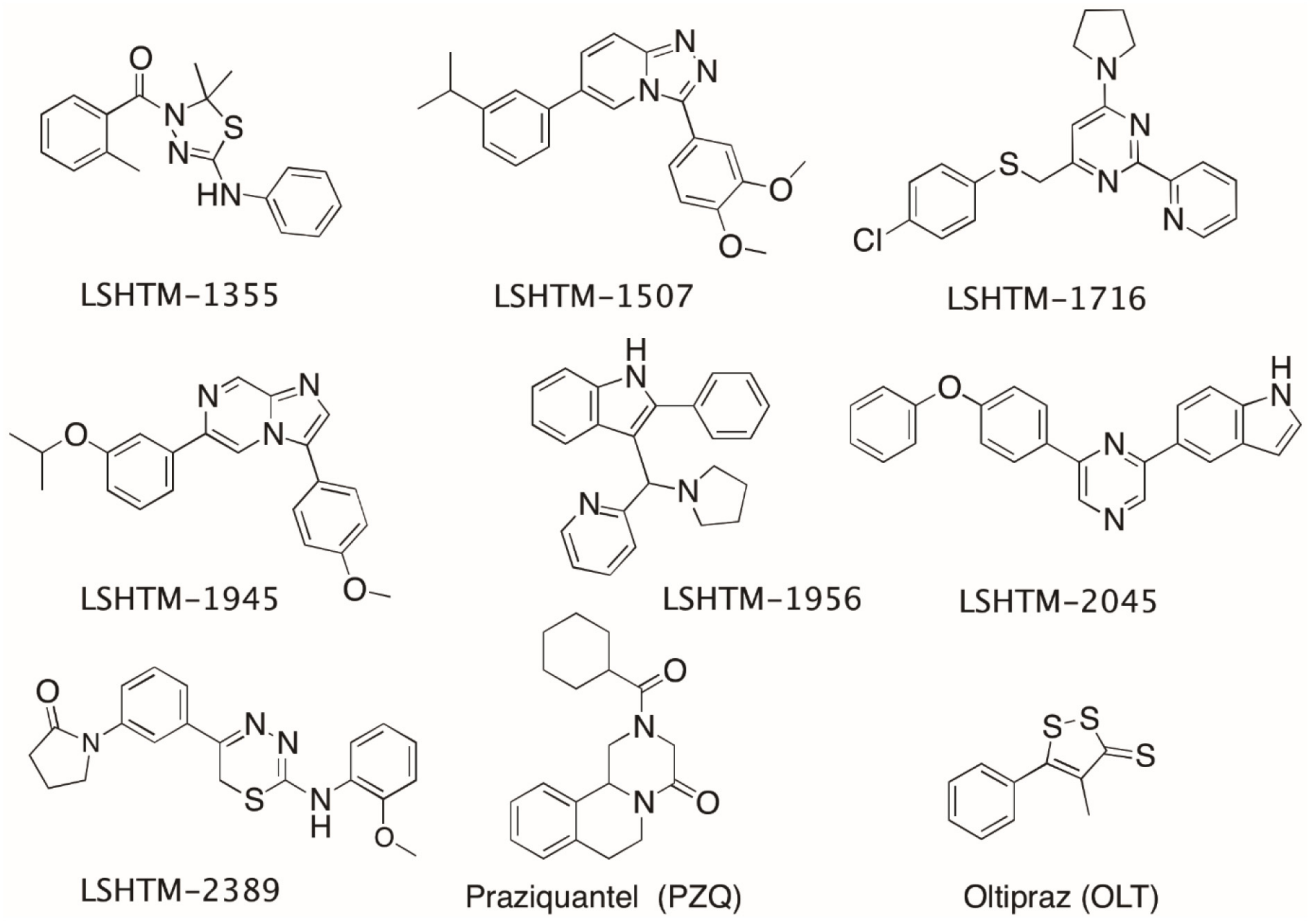
Chemical structures of standard compounds and seven priority compounds selected for further follow up.

Following the initial screening cascade and subsequent IC50 testing, seven compounds were selected for further follow up on the basis of balanced activity against all parasite life stages, a good cytotoxicity window and structural attractiveness. Additional hits with less compelling profiles were also identified and are shown in S1 Table. The structures of the seven priority compounds and two standards (PZQ and OLT) which we have used in our HTS assays are shown in Fig 3 and the activity is described in Table 3. Activity of all compounds was confirmed with solid sample which was purchased commercially if available, or re-synthesised. The activity of solid samples was in good agreement with the original liquid sample data. It was encouraging that the screening process was able to identify multiple different chemotypes with similar potency to the standard agent (PZQ) against juvenile worms.

**Table 3:** Activity data for compounds of interest.

CompoundID (LHSTM-)	ALogP^	Larval EC50 (μM)	Juvenile EC50 (μM)	Adult EC50 (μM)	MRC5 CC50 (μM)
Praziquantel	2.5	1.9	4.7	0.3	>50
Oltipraz	2.2	0.5	0.8	0.85	>50
1355	3.9	6.7	5.8 (*14*.3*)	5.3* (*11*.2*)	>50 (33.8)
1507	4.9	6.6	4.0 *(9*.8*)	1.6* (*3*.0*)	18.6
1945	4.0	6.7	5.7 (*13*.7*)	4.9*	40.1
1956	5.3	0.5	2.5 (*3*.7*)	3.2* (*11*.8*)	86.0
2045	5.3	1.4	2.1 (*3*.6*)	3.7* (*11*.0*)	20.0
2389	3.3	6.1	5.1 *(5)*	6.0* *(7*.1*)	>50 *(>50)*
1716	5.1	0.3	11.0 (*9*.*5*)	2.8* (*5*.4*)	>50 (>50)

Potency data are geometric means of at least two values except compounds indicated with * (n = 1). Numbers in italics were obtained from original liquid sample data.

^^^AlogP calculated using BioVia’s ScienceCloud (https://www.sciencecloud.com/about/index.html)

All of the seven hit molecules comply with the Lipinski rules-of-five for oral absorption as originally defined [17]. Some of the compounds failed the lipophilicity criteria (ALogP >5) but Lipinski rules predict that this single failure is unlikely to significantly impair absorption.

We were interested to know the speed of action of the selected compounds, therefore activity was examined in juvenile and adult worm assays at both 1 day and day 5. The data in Table 4 shows that most of the compounds of interest had more rapid and complete activity against juvenile worms than PZQ which only achieved 58.4% motility reduction after 5 days. Two compounds, 2389 and 1716 appeared to have a delayed action with no inhibition after 1 day, but complete inhibition after 5 days. The effects of compounds on adult worms were generally similar to PZQ.

**Table 4:** Inhibition of worm motility at day 1 and day 5.

Compound ID	Juvenile (% motility reduction)		Adult % motility reduction	
	DAY 1	DAY 5	DAY 1	DAY 5
PZQ	57	58	76	88
OLT	51	100	50	95
1355	75	75	75	100
1507	75	75	75*	88*
1945	75	75	75*	75*
1956	75	100	56	81
2045	50	100	37	88
2389	0*	100*	12.5	100
1716	0	84	64	98

All compounds were tested at 12.5μM. Data are geometric means of at least two values except compounds indicated with * (n = 1).

### Analysis of Publicly Available Data Related to the Seven Most Promising Compounds

The choice of series to move forward for further chemical optimisation is a crucial one in the life of any small molecule drug discovery project. Multiple factors are likely to affect ultimate success of the project including: physicochemical properties, presence of structural groups linked with toxicity, synthetic tractability, freedom to operate from an IP perspective. One of the factors affecting the decision which is sometimes overlooked is to understand what is known about existing pharmacology of the hit or compounds like it. To that end we have followed a similar process to that described by Avery *et al* [18]. The compounds listed in Table 2 were subjected to an analysis using a freely available workflow based on KNIME (http://www.knime.org/downloads/overview), ChEMBL (doi:10.1093/nar/gkr777) and DataWarrior (http://www.openmolecules.org/datawarrior/download.html) [25]. called ‘Know Your Molecule’. The workflow is described and can be downloaded from (http://www.mmv.org/research-development/computational-chemistry).

A table of the findings from this is provided in S2 Table. This data indicates for example that close analogues of LSHTM-1956 have been shown to have activity against S. mansoni peroxiredoxin Prx2 at a range of potencies 100–1μM [19, 20]. Although these analogues possess a methyl at the 2-position of the indole rather than a phenyl, it is an interesting mechanistic link to explore.

Close analogues of LSHTM-1716 have shown activity in a variety of whole cell assays, but simplified analogues appear less promiscuous. Simpler analogues of LSHTM-2045 were reported active against *P*. *falciparum* at 600–750nM and released by GSK as part of the TCAMS. Encouragingly both were also recorded as inactive (7% and 9% inhibition at 10μM) in a HepG2 cell line cytotoxicity. Finally, two close analogues of LSHTM-1586 are recorded as showing activity below 1μM in 9 separate screens, perhaps supporting the finding in our hands that the resynthesized compound was cytotoxic (S1 Table). Although no further work has been done to confirm the reported activities, some of the data can be useful signposts to help prioritise the hits and to identify hypotheses to test in subsequent optimisation.

## Discussion

Based on the need to develop new drugs for improved treatment of schistosomiasis, we carried out a high throughput screening campaign with the aim of discovering compounds with balanced potency against all life stages of *Schistosoma mansoni*. We identified a number of novel compounds of interest and have prioritised seven of these for further follow up.

We were fortunate to be able to access high quality libraries from pharma companies and other sources for this project. Surprisingly the hit rates for each compound collection were markedly different, with the Pfizer and GSK sets having significantly higher hit rates than the other libraries. There are two notable differences between compound collections. Firstly the GSK and Pfizer subsets have higher mean lipophilicity and molecular weight compared to the other collections. Secondly, the MMV and Enamine collections are diversity screening libraries, whereas the Pfizer and GSK libraries both comprise compounds which have previously been reported to have activity against one or more pathogens. Both the physicochemical property differences and different selection biases might be expected to influence hit rate in a new phenotypic screen such as this.

Our ability to screen nearly 300,000 compounds in a short time was due to use of a high throughput larval screen [11]. To efficiently progress the hits from this screen we developed and implemented a screen using juvenile (21 day) worms as we were particularly keen to identify compounds with improved drug efficacy against this stage of the parasite life cycle. The juvenile assay proved to be a very efficient medium throughput screen which was very efficient at triaging compounds prior to testing in the adult worm assay. It is encouraging that we have been able to identify a number of compounds with IC50 <10 μM against all stages of the parasite life cycle and with potency against juvenile worms comparable with praziquantel. Typically, medicinal chemistry optimisation of HTS hits can result in early leads that are >10-fold more potent [21] and advanced leads that are 100-1000-fold more potent than the original hit. A similar potency improvement was also achieved during the discovery process leading to praziquantel [22]. Therefore we are optimistic that medicinal chemistry optimisation of our hits could lead to a candidate superior to praziquantel.

It is interesting to note that we identified two compounds (1507 and 1716) which like PZQ are more potent against the adult stage than juveniles, whereas a number of other compounds showed comparable activity against both stages as was also the case for oltipraz, a compound which has proved to be effective in humans but whose development and use clinically was halted due to phototoxicity [23, 24]. This may suggest that the targets of these compounds are differentially expressed, or play greater or lesser roles in the different life stages of the parasite.

Investigation of compound activity at two different time points revealed that several of our compounds appear to cause faster and more complete inhibition of juvenile worm motility compared with PZQ. It will be interesting to explore if this translates to faster cures in an *in vivo* efficacy model. The lower effect of PZQ on juvenile compared with adult motility may also explain the relatively poor clinical efficacy of this compound against juvenile versus adult worms.

All seven of our priority compounds comply with the Lipinski rules-of-five for oral absorption [17] making them attractive starting points for further medicinal chemistry optimisation. This is particularly important as oral treatment is standard for schistosomiasis. Analysis of the structures of our seven priority compounds reveals that 1355 and 2389 have related structures, as do 1507 and 1945. This may indicate that these pairs of compounds could have similar modes-of-action, although the mechanism of action of all our hits remain unknown at this time.

To the best of our knowledge, there are no new compounds in late pre-clinical or clinical development for schistosomiasis, therefore the molecules we have identified represent an exciting starting point for drug discovery. We are currently working to optimise selected series from our hit set with the initial goal of making analogues with pharmacokinetic properties that would allow demonstration of *in vivo* activity and ultimately with the aim of identifying a clinical candidate.

## Supporting Information

S1 Table Table of additional actives obtained during the screening. These structures were deprioritised relative to those in the body of the paper for the reasons given in the comment column. The assays are those described in the paper. ND = No data obtained.
(XLSX)

S2 Table This table lists each of the structures in the paper (and a couple described in S1 Table) and then a summary of findings obtained using the KNIME workflow ‘Know Your Molecule’ to access and present ChemBl data as described in the paper. The structures shown are those with similarity to the compound number listed in column 1 of the table.
(XLSX)
